# Serine 89 Phosphorylation Controls Nuclear Localization and Transcriptional Activity of ARID3B

**DOI:** 10.3390/cells15070612

**Published:** 2026-03-30

**Authors:** Micneya Landeros-Rodriguez, Krishna Ailiani, Richard Dahl, Karen D. Cowden Dahl

**Affiliations:** 1 Kabara Cancer Research Institute, Gundersen Medical Foundation, 1300 Badger Street, La Crosse, WI 54601, USA; micneya.landerosrodriguez@emplifyhealth.org (M.L.-R.); krishnaailiani@gmail.com (K.A.); 2 Department of Microbiology and Immunology, Indiana University School of Medicine, South Bend, IN 46617, USA; richdahl@iu.edu

**Keywords:** ARID3B, phosphorylation, transcriptional regulation, ovarian cancer, glioblastoma

## Abstract

Transcription factors that control stem cell programs are central drivers of cancer progression, metastasis, and therapy resistance. ARID3B, a DNA-binding protein overexpressed across multiple tumor types, expands the cancer stem cell population by regulating these pathways. Yet, how ARID3B is regulated remains largely unknown. Here, we uncover phosphorylation at Serine 89 as a critical switch controlling ARID3B localization and function. We used site-directed mutagenesis to generate phospho-dead (S89A) and phospho-mimetic (S89D) ARID3B constructs, and we generated a phospho-specific antibody for S89. With these tools, we showed that phosphorylation confines ARID3B to the nucleus in ovarian cancer and glioblastoma cells, as well as in human tissues, while unphosphorylated ARID3B can localize to the nucleus, cytoplasm, and membrane. Functionally, S89D mirrors wild-type ARID3B in regulating key transcriptional programs, whereas S89A diverges, consistent with altered subcellular localization. Chromatin immunoprecipitation confirms that direct gene regulation is enhanced in WT ARID3B and S89D compared to cells expressing S89A. Collectively, these findings reveal phosphorylation as a previously unrecognized molecular switch that dictates ARID3B’s localization and transcriptional activity, providing novel insights into cancer stem cell regulation and identifying a potential targetable vulnerability in aggressive tumors.

## 1. Introduction

The AT-Rich Interactive Domain (ARID) family of DNA-binding proteins is involved in chromatin remodeling and the regulation of gene expression. Members of this family are characterized by the ARID DNA-binding domain, a highly conserved sequence of ~100 amino acids [1]. Among these proteins, particularly in cancer, ARID3B is an important regulator of stem cell-associated gene expression. The first indication that ARID3B may provide a role in stemness came from a proteomics screen that found ARID3B protein in NAC1-containing complexes in embryonic stem cells [2]. Additional support for a role in stemness came when it was demonstrated that ARID3B increases during iPS reprogramming [3]. We subsequently demonstrated that ARID3B increases the production of ovarian cancer stem cells (CSCs) and activates a stem cell gene signature [4,5]. In agreement with our work, ARID3B was shown to regulate stem cell genes in oral and head and neck cancers [6]. Collectively, these studies establish ARID3B as a transcriptional regulator of cancer stem cell programming.

ARID3B is overexpressed in a number of types of malignancies, including neuroblastoma, ovarian, head and neck, and breast [6,7,8,9]. However, the overexpression of ARID3B in cancer is unlikely to be driven by mutation. Analysis of cBioPortal data identified 91 mutations in ARID3B across nineteen tumor types. Thirteen tumor types had amplifications in ARID3B. Importantly, point mutations were found throughout the entire ARID3B gene with no apparent hotspots. Moreover, only seven of the mutations appeared in multiple tumors. These analyses suggest that while somatic mutations in ARID3B are rare (0.8% frequency), alternative regulatory mechanisms may contribute to its elevated expression in cancer. Importantly, elevated ARID3B expression was shown to correlate with poorer clinical outcomes. Higher expression of ARID3B correlated with decreased overall survival for patients with blood, brain, breast, and skin cancers [6]. In addition, moderate nuclear ARID3B expression correlated with decreased time until relapse in serous ovarian cancer [5]. Moreover, preclinical studies demonstrate that ARID3B promotes tumor growth (in neuroblastoma, ovarian cancer, and head and neck cancer) [5,6,7]. Therefore, the in vivo data in patients and animal studies strongly suggest a role for ARID3B in promoting tumor progression.

The primary mechanism described for increasing ARID3B expression and/or activity is post-transcriptional regulation via microRNAs, including let-7 and miR-125a [6]. Loss of miRNAs in cancer may lead to increased ARID3B and subsequent activation of ARID3B transcriptional targets. While miRNA-mediated regulation may explain the increased abundance of ARID3B protein, the regulation of ARID3B targets is more complex. Notably, the post-translational regulation of ARID3B function has not been explored. To further understand the regulation of ARID3B in cancer, we focused on phosphorylation. According to PhosphoSite.org, ARID3B can be phosphorylated on 21 distinct residues, with Serine 89 being the most frequently reported site. Several of the studies cited on PhosphoSite.org examined phosphorylation in both cancer and stem cells. Therefore, we hypothesize that phosphorylation of ARID3B at serine 89 is critical in the regulation of stem cell-associated genes and cancer-associated phenotypes.

## 2. Materials and Methods

### 2.1. Cell Culture

All cell lines were maintained at 37 °C in a humidified incubator with 5% CO_2_. OVCA429 and OVCA433 cells were provided by Dr. Bast (MD Anderson Cancer Center, Houston, TX, USA) [10]. U87MG cells were purchased from the American Type Culture Collection (ATCC, Manassas, VA, USA). OVCA429, OVCA433, and U87MG cells were cultured in Minimum Essential Medium (MEM; Thermo Fisher Scientific, Waltham, MA, USA) supplemented with 10% fetal bovine serum (FBS; Peak Serum, Wellington, CO, USA), 0.1 mM L-glutamine, 1 mM sodium pyruvate, 50 U/mL penicillin, and 50 μg/mL streptomycin (Thermo Fisher Scientific, Waltham, MA, USA).

In addition, 293FT cells were purchased from Thermo Fisher Scientific (Waltham, MA, USA) and cultured in Dulbecco’s Modified Eagle Medium (DMEM; Thermo Fisher Scientific, Waltham, MA, USA). DMEM was supplemented with 10% FBS (Sera Prime, Fort Collins, CO, USA), 0.1 mM L-glutamine, 1 mM sodium pyruvate, 50 U/mL penicillin, and 50 μg/mL streptomycin. OVCAR4 cells were purchased from MilliporeSigma (Burlington, MA, USA) and cultured in RPMI-1640 supplemented with 10% FBS (Sera Prime, Fort Collins, CO, USA), 2 mM L-glutamine, 0.25 U/mL insulin, 50 U/mL penicillin, and 50 μg/mL streptomycin. All tissue culture additives were obtained from Thermo Fisher Scientific (Waltham, MA, USA). OVCA429, OVCA433, and U87MG cells were lentivirally transduced with constructs encoding green fluorescent protein (GFP), ARID3B-GFP, or the phosphorylation mutants S89A or S89D (mutated from ARID3B-GFP; GeneCopoeia, Rockville, MD, USA).

### 2.2. RNA-Sequencing (RNA-Seq)

RNA-seq was performed on OVCA429 cells expressing GFP, ARID3B-GFP (WT), S89A, or S89D. Total RNA was isolated from three independent biological replicates per condition using TRIzol reagent (Thermo Fisher Scientific) according to the manufacturer’s instructions. RNA purity and integrity were assessed by spectrophotometric analysis using A260/280 and A260/230 ratios prior to library preparation. Poly(A)-enrichment, RNA libraries, and RNA-sequencing were performed by Novogene (Sacramento, CA, USA). RNA integrity was assessed using the Bioanalyzer 2100 system (Agilent Technologies, Santa Clara, CA, USA). First, a non-strand-specific library was generated. Messenger RNA was purified from total RNA using poly-T oligo-attached magnetic beads. After fragmentation, the first strand cDNA was synthesized using random hexamer primers, followed by the second strand cDNA synthesis. The library was ready after end repair, A-tailing, adapter ligation, size selection, amplification, and purification. The library was checked with Qubit and real-time PCR for quantification and a bioanalyzer for size distribution detection. Then, Strand specific library was constructed. Messenger RNA was purified from total RNA using poly-T oligo attached magnetic beads. After fragmentation, the first strand cDNA was synthesized using random hexamer primers. Then, the second strand cDNA was synthesized using dUTP instead of dTTP. The directional library was ready after end repair, A-tailing, adapter ligation, size selection, amplification, and purification. The library was checked with Qubit and real-time PCR for quantification and a bioanalyzer for size distribution detection. Next, clustering and sequencing were performed. After library quality control, different libraries were pooled based on the effective concentration and targeted data amount, and then subjected to Illumina sequencing. The basic principle of sequencing is “Sequencing by Synthesis”, where fluorescently labeled dNTPs, DNA polymerase, and adapter primers are added to the sequencing flow cell for amplification. As each sequencing cluster extends its complementary strand, the addition of each fluorescently labeled dNTP releases a corresponding fluorescence signal. The sequencer captures these fluorescence signals and converts them into sequencing peaks through computer software, thereby obtaining the sequence information of the target fragment. Following the sequencing was the bioinformatics analysis pipeline. Data quality control was initiated. Raw data (raw reads) of fastq format were first processed through the fastp software. In this step, clean data (clean reads) were obtained by removing reads containing adapters, reads containing poly-N, and low-quality reads from the raw data. At the same time, Q30 and GC content of the clean data were calculated. All the downstream analyses were based on the clean data with high quality. Reference genome and gene model annotation files were downloaded from the genome website directly. An index of the reference genome was built using Hisat2 v2.0.5, and paired-end clean 1 reads were aligned to the reference genome using Hisat2 v2.0.5. We selected Hisat2 as the mapping tool because it can generate a database of splice junctions based on the gene model annotation file, and thus, a better mapping result than other non-splice mapping tools. Quantification of gene expression level was conducted via featureCounts v1.5.0-p3, which was used to count the reads numbers mapped to each gene. And then, the FPKM of each gene was calculated based on the length of the gene and reads count mapped to this gene. FPKM (Fragments Per Kilobase of transcript per Million mapped reads) was calculated to normalize for sequencing depth and gene length. Next was differential expression analysis. For DESeq2 with biological replicates: differential expression analysis for two conditions/groups was performed using the DESeq2 R package (1.20.0). DESeq2 provides statistical programs for determining differential expression in digital gene expression data using models based on the negative binomial distribution. The resulting *p*-value is adjusted using Benjamini and Hochberg’s methods to control the error discovery rate. The corrected *p*-value ≤ 0.05 and |log2(foldchange)| ≥ 1 were set as the threshold of significant differential expression. For edgeR without biological replicates: prior to differential gene expression analysis for each sequencing library, read counts were adjusted using the edgeR R package (3.22.5) by scaling normalization factors to eliminate differences in sequencing depth between samples, followed by differential expression analysis. The resulting *p*-value is adjusted using Benjamini and Hochberg’s methods to control the error discovery rate. The corrected *p*-value ≤ 0.05 and |log2(foldchange)| ≥ 1 were set as the threshold of significant differential expression. Finally, GO and KEGG enrichment analyses of differentially expressed genes were conducted. Gene Ontology (GO) enrichment analysis of differentially expressed genes was implemented by the clusterProfiler R package, in which gene length bias was corrected. GO terms with corrected *p*-value less than 0.05 were considered significantly enriched by differentially expressed genes. KEGG is a database resource for understanding high-level functions and utilities of the biological system, such as the cell, the organism, and the ecosystem, from molecular-level information, especially large-scale molecular datasets generated by genome sequencing and other high-throughput experimental technologies (http://www.genome.jp/kegg/). We used the clusterProfiler R package to test the statistical enrichment of differentially expressed genes in KEGG pathways. The Reactome database brings together the various reactions and biological pathways of the human model species. Reactome pathways with corrected *p*-values less than 0.05 were considered significantly enriched by differentially expressed genes. The DO (Disease Ontology) database describes the function of human genes and diseases. DO pathways with corrected *p*-values less than 0.05 were considered significantly enriched by differentially expressed genes. The DisGeNET database integrates human disease-related genes. DisGeNET pathways with corrected *p*-values less than 0.05 were considered significantly enriched by differentially expressed genes. We used clusterProfiler software to test the statistical enrichment of differentially expressed genes in the Reactome pathway, the DO pathway, and the DisGeNET pathway.

### 2.3. Whole Cell Lysates, Subcellular Fractionation, and Nuclear and Cytoplasmic Extracts

Whole cell lysates were prepared by lysing OVCA429 and U87MG cells in RIPA buffer (50 mM Tris pH 7.5, 150 mM NaCl, 1% NP-40, 0.5% EDTA, and 1× Halt Protease Inhibitor Cocktail and 1× Phosphatase inhibitor (Pierce, Rockford, IL, USA). Protein concentration was determined using bicinchoninic acid (BCA) assay according to the manufacturer’s protocol (Pierce Biotechnology, Rockford, IL, USA). OVCA429 cells expressing GFP, ARID3B-GFP, S89A, or S89D were grown on multiple 10 cm plates. Cells were fractionated according to the Subcellular Protein Fractionation Kit purchased from (Thermo Fisher Scientific, Waltham, MA, USA). Similarly, the NE-PER Nuclear and Cytoplasmic Extraction Kit (Thermo Fisher Scientific, Waltham, MA, USA) was used to isolate nuclear and cytoplasmic protein fractions. Lysates were separated on SDS-PAGE gels.

### 2.4. Immunoprecipitation (IP)

Immunoprecipitation assays were conducted on nuclear and cytoplasmic fractions from OVCA429 cells expressing GFP, ARID3B-GFP, S89A, or S89D. GFP-TRAP agarose was used to immunoprecipitate GFP fusion proteins according to the manufacturer’s instructions (Bulldog Bio, Portsmouth, NH, USA).

Briefly, protein concentrations of extracts were determined using a BCA kit (Pierce Biotechnology, Rockford, IL, USA). GFP-TRAP agarose beads were washed in RIPA buffer with 1× Halt Protease Inhibitor Cocktail and 1× Phosphatase inhibitor (Pierce, Rockford, IL, USA). For each IP, 25 mL of bead slurry was combined with 1 mg of protein extract and rotated at 4 °C for 1 h. Beads were washed with RIPA buffer three times and resuspended in 2× Laemmli Buffer prior to boiling. IPed complexes were resolved by SDS-PAGE. Ten percent input samples (100 mg of each extract) were included and separated in parallel.

### 2.5. Western Blots

Whole-cell lysates, subcellular fractions, nuclear and cytoplasmic extracts, and IPs were separated by SDS-PAGE and transferred to nitrocellulose for immunoblotting. Proteins were detected using antibodies against ARID3B (Fortis Life Sciences, Boston, MA, USA), GAPDH (Cell Signaling Technology, Danvers, MA, USA), Integrin β1 (Fortis Life Sciences, Boston, MA, USA), Histone H3 (Cell Signaling Technology, Danvers, MA, USA), ARID3A (Active Motif, Carlsbad, CA, USA), and EGFR (Cell Signaling Technology, Danvers, MA, USA), followed by HRP-conjugated anti-rabbit secondary antibodies (Cell Signaling Technology, Danvers, MA, USA). Each experiment that resulted in Western blotting was conducted independently, a minimum of three times. Representative blots from one experiment are shown.

A phospho-specific polyclonal antibody recognizing serine 89 of ARID3B was custom-generated (Sino Biological, Wayne, PA, USA). Signal detection was performed using a ChemiDoc Touch Imaging System with Image Lab software Version 6.1(Bio-Rad Laboratories, Hercules, CA, USA).

### 2.6. Fluorescence

Localization of ARID3B-GFP fusion proteins was detected, and images were collected via live fluorescence imaging on an EVOS M5000 microscope (Invitrogen, Carlsbad, CA, USA). To examine the expression and localization of phospho-S89 ARID3B and ARID3B, cells were plated on 4-well chamber slides. Cells were fixed with ice-cold acetone. Cells were permeabilized with Triton X-100 and blocked with 3% bovine serum albumin (BSA) in Tris-buffered saline with 0.1% Tween-20 (TBST). Cells were incubated with appropriate primary antibodies (phospho-S89 ARID3B or ARID3B (Fortis Life Sciences)), followed by anti-mouse Alexa Fluor 594 secondary (Thermo Fisher, Waltham, MA, USA). Slides were mounted with Fluoromount G with DAPI (Thermo Fisher, Waltham, MA, USA). Images were collected using an EVOS M5000 (Invitrogen, Carlsbad, CA, USA). Immunofluorescence and live cell imaging were each conducted >3 times. To assess nuclear/cytoplasmic expression of GFP, WT, S89A, and S89D, GFP distribution was counted from 3 separate fields from 3 independent transductions. All of the cells in each field were counted. Then, the number of cells with both nuclear and cytoplasmic GFP expression, only nuclear, or only cytoplasmic GFP was counted. The distribution is represented as a percentage of the total in Supplemental Figure S1.

### 2.7. RT-qPCR

Total RNA was isolated via TRIzol reagent according to the manufacturer’s protocol (Thermo Fisher, Waltham, MA, USA). Complementary DNA (cDNA) was generated from 2 mg of RNA using the High-Capacity cDNA Reverse Transcription Kit (Life Technologies, Waltham, MA, USA). Reverse transcribed quantitative polymerase chain reaction (RT-qPCR) was conducted for: NES, PROM1, TNF, TNFRSF1B, WNT4, MYCN, and PROM2. Reactions were run either using SsoFast EvaGreen Supermix or SsoAdvanced Universal Probes Supermix (Bio-Rad Laboratories, Hercules, CA, USA). All gene expression primer sets were obtained from Integrated DNA Technologies (Coralville, IA, USA), see Supplementary Table S1. RT-qPCR reactions were run in triplicate and normalized to GAPDH expression. RNA collections were conducted at least three times.

### 2.8. Immunohistochemistry (IHC)

Human tissue microarrays were purchased from TissueArray.com LLC (Derwood, MD, USA). IHC was performed on a paraffin-embedded normal human tissues microarray BN243d, which contained duplicate cores. Staining for phosphorylated ARID3B and total ARID3B was performed using the VECTASTAIN ABC Kit with ImmPACT DAB substrate and Hematoxylin QS counterstain (Vector Laboratories, Burlingame, CA, USA). Anti-ARID3B IHC antibody was obtained from Fortis Life Sciences (Montgomery, TX, USA), and the custom phospho-ARID3B antibody was used for the detection of serine 89 phosphorylation.

### 2.9. Chromatin Immunoprecipitation (ChIP)

Chromatin immunoprecipitation was performed on three independent collections of chromatin from OVCA429 cells expressing GFP, ARID3B-GFP, S89A, or S89D using the Imprint Chromatin Immunoprecipitation Kit (Sigma-Aldrich, St. Louis, MO, USA), following the manufacturer’s instructions. Cells were trypsinized, crosslinked with formaldehyde, quenched, and washed, and nuclei were isolated. Chromatin was sheared by sonication. Protein concentrations were determined by BCA assay to ensure equal input for immunoprecipitations. Immunoprecipitations were performed using GFP-Trap agarose (Bulldog Bio, Portsmouth, NH, USA) or normal rabbit IgG as a control. After washing, crosslinks were reversed, and DNA was purified. qPCR was performed at an ARID3B binding site located at chr17:79917005–79917199 in TNF. ChIP and input DNA were analyzed by quantitative PCR (qPCR) using locus-specific primer sets (amplicon length 195 bp). Reactions were performed using Sso Fast EvaGreen Supermix (Bio-Rad Laboratories, Hercules, CA, USA).

### 2.10. ChIP Quantitation and Data Analysis

ChIP enrichment was quantified using the percent input (% Input) method. Input DNA was processed in parallel with immunoprecipitated samples and analyzed by qPCR. Quantification cycle (Cq) values were obtained using automatic threshold settings. For each locus, percent input was calculated using the following formula:

% Input = 100 × 2^(Cqinput−CqChIP)^

Percent input values were calculated for both target antibody immunoprecipitations, IgG negative controls (Invitrogen, Carlsbad, CA, USA), and H3K4me (for active chromatin) (Abcam, Waltham, MA, USA). Fold change was calculated relative to the OVCA429 GFP. Three replicates were averaged prior to downstream calculations. Statistical analyses were performed using biological replicates, and data are presented as mean ± SEM. Statistical significance was assessed using Student’s *t*-tests (GraphPad Prism software (version 10.6 for macOS)), as indicated in the figure legends.

### 2.11. Statistics

RT-PCR gene expression data were analyzed using GraphPad Prism software (version 10.6 for macOS). Relative expression values (relative to untransfected parental cells or GFP-expressing cells) from three biological samples per group were included in the analysis. Comparisons among multiple groups were performed using one-way ANOVA. When a significant overall effect was detected, Tukey’s multiple comparisons test was applied to assess pairwise differences between groups while controlling for multiple testing. Adjusted *p*-values < 0.05 were considered statistically significant.

## 3. Results

ARID3B was originally described as an exclusively nuclear transcription factor, while its paralog, ARID3A, shuttles between the nucleus and cytoplasm [6,11]. However, we reported that exogenous ARID3B can localize to both the nucleus and cytoplasm. Notably, following lentiviral transduction into cancer cells, ARID3B is initially found in the nucleus, but localization becomes increasingly cytoplasmic with prolonged culture [12]. Additionally, ARID3B is detected in both the nucleus and the cytoplasm in human tissues; however, the extent of nuclear localization varied by tissue [8]. Together, these observations suggested that post-translational mechanisms may regulate the subcellular localization of ARID3B.

**Figure 1:**
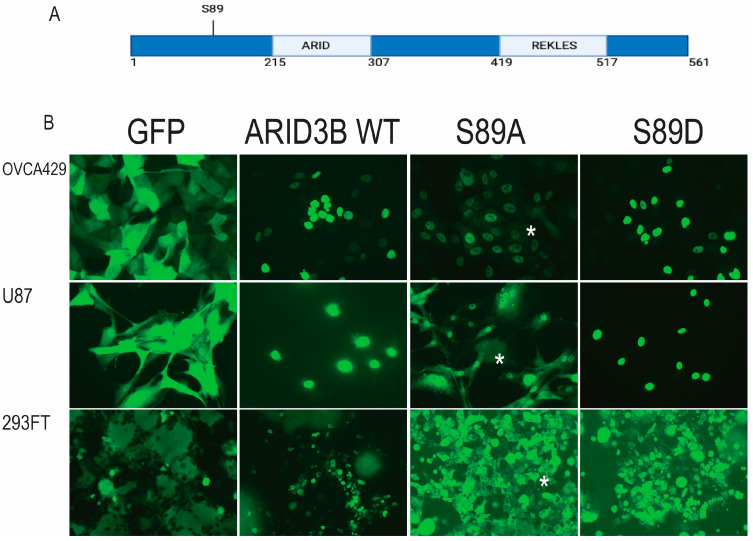
Subcellular localization of ARID3B-GFP constructs in live cells. (**A**) Schematic representation of the ARID3B protein highlighting functional domains and the location of serine 89 (S89). (**B**) Live-cell fluorescence microscopy of OVCA429, U87MG, and 293FT cells expressing GFP alone, wild-type (WT) ARID3B–GFP, ARID3B–GFP S89A, or ARID3B–GFP S89D, demonstrating construct-dependent differences in subcellular localization. Live cell imaging on independently transduced or transfected (293FT) cells has been conducted >3 times. Green = expression of GFP or GFP-fusion proteins. * = cytoplasmic localization of S89A.

Since phosphorylation is a common method for modulating subcellular localization, we examined whether ARID3B is phosphorylated. PhosphoSite.org identified serine 89 (S89) as the most frequently reported putative phosphorylation site, with additional potential sites at S165 and S381. Interestingly, none of these residues are located in the ARID or REKLES domains (Figure 1). To assess the functional role of S89 phosphorylation, we performed site-directed mutagenesis (SDM) of a lentiviral ARID3B-GFP expression construct, generating a phospho-dead mutant (S89A) and a phosphomimetic (S89D). We transduced ovarian cancer OVCA429 and U87MG glioblastoma cells with GFP, wild-type (WT) ARID3B, ARID3B S89A, and ARID3B S89D. We also examined 293FT cells transfected with GFP, WT, S89A, and S89D. Live-cell fluorescence imaging of the GFP-fusion proteins revealed predominant nuclear localization of WT and S89D. S89A was detected both in the cytoplasm and nucleus (Figure 1B and Figure S1). We acknowledge that overexpression of proteins has limitations. Importantly, when proteins are overexpressed at non-physiological levels, it is hard to discern if phenotypic changes have biological relevance. We found that overexpression of ARID3B ranged from Log_2_ fold change of 2.5 to 3.75. While the ARID3B constructs are clearly overexpressed, the magnitude of ARID3B is not unreasonable, given that many tissues, like ovarian surface epithelium or the cerebellum, do not appear to express much ARID3B protein, and others, like skin and colon, have high ARID3B expression [8].

**Figure 2:**
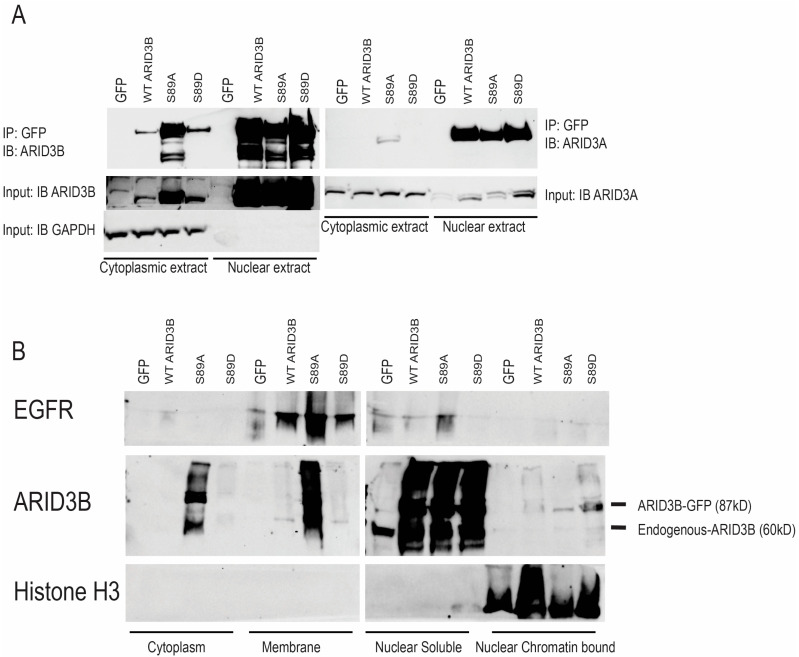
Biochemical analysis of ARID3B subcellular localization. (**A**) Nuclear and cytoplasmic fractions were isolated from OVCA429 cells expressing GFP, WT ARID3B–GFP, S89A, or S89D. GFP immunoprecipitations (IPs) were performed, and IPs and input lysates were analyzed by immunoblot (IB) (Western blot) for ARID3B (left) and ARID3A (right). GAPDH served as a loading control for input samples and a marker for the cytoplasmic fraction. IPs were conducted on 3 independent fractionations, and one representative experiment is shown. (**B**) Subcellular fractionation of OVCA429 cells expressing GFP, WT, S89A, or S89D, followed by Western blot analysis for ARID3B. EGFR and Histone H3 were used as membrane and nuclear chromatin-bound markers, respectively, to confirm fractionation quality. Independent transductions and fractionations were subjected to Western blot analysis thrice, with one representative experiment shown.

The imaging results were validated biochemically by isolation of nuclear and cytoplasmic extracts from OVCA429 cells, followed by immunoprecipitation (IP) using the GFP tag and immunoblot (Western blot) (IB) (Figure 2A). WT, S89A, and S89D were abundant in nuclear fractions when immunoprecipitated GFP fusion proteins were blotted for total ARID3B. Additionally, S89A was also highly expressed in the cytoplasmic fractions. Immunoprecipitations were subsequently blotted for the ARID3B paralog, ARID3A (Figure 2A, right panel). WT, S89A, and S89D were all associated with the ARID3B paralog, ARID3A, in nuclear fractions. Therefore, phosphorylation status alone does not impact heterodimerization. S89A showed minimal ARID3A association in the cytoplasm. Next, we conducted subcellular fractionation on OVCA429 cells expressing GFP, WT, S89A, and S89D, followed by a Western blot for total ARID3B (Figure 2B). In agreement with the data in Figure 2A, S89A is found in cytoplasmic, membrane, nuclear-soluble, and nuclear chromatin-bound (containing Histone H3) extracts. WT and S89D were abundant in the nuclear-soluble fraction, and S89D showed the strongest association with chromatin. These initial studies demonstrate that phosphorylation of ARID3B at S89 is critical to its nuclear localization and/or retention.

**Figure 3:**
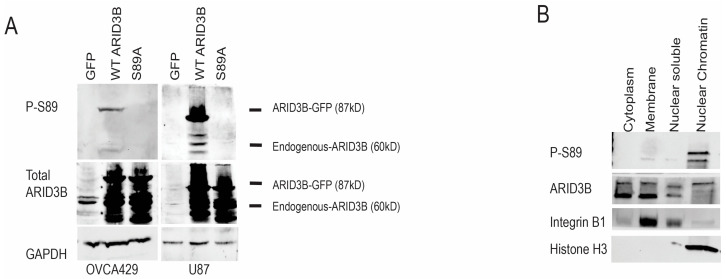
Phosphorylation of ARID3B at S89 is associated with nuclear localization. (**A**) Whole-cell lysates from OVCA429 and U87MG cells expressing GFP, WT ARID3B–GFP, or S89A were analyzed by Western blot using a phospho-specific antibody against ARID3B S89 (P-S89), total ARID3B, and GAPDH (loading control). Locations of endogenous (60 kD) and overexpressed ARID3B (87 kD) are shown. (**B**) Subcellular fractionation of OVCA429 cells, followed by Western blot analysis for endogenous P-S89 ARID3B, total ARID3B, Integrin β1 (cytoplasmic marker), and Histone H3 (nuclear chromatin-bound fraction marker). Three different fractionations yielded similar results.

**Figure 4:**
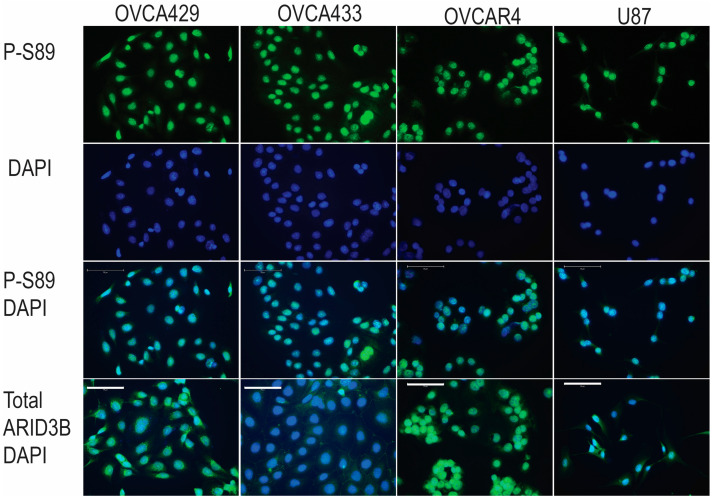
ARID3B localizes to nuclei. Immunofluorescence staining of OVCA429, OVCA433, OVCAR4, and U87MG cells for phospho-S89 ARID3B and total ARID3B demonstrates nuclear enrichment of total and phosphorylated ARID3B. DAPI staining denotes nuclei. IF staining has been conducted >3 times with similar results. Scale bar = 75um.

While the phosphorylation mutants are important tools to study ARID3B phosphorylation, we also wanted to assess endogenous phosphorylation. We generated a polyclonal antibody (from Sino Biological) directed against phosphorylated S89 in ARID3B. We first validated the specificity of the antibody via Western blot analysis. We isolated whole cell lysates from OVCA429 and U87MG cells expressing GFP, WT ARID3B, and S89A (where the fusion protein cannot be phosphorylated). Lysates were separated on an SDS-PAGE gel. Western blot identified a specific band ~90 kD (the molecular weight of 60 kD ARID3B fused to 27 kD GFP) consistent with the ARID3B-GFP fusion. The 90 kD band was only detected in WT-expressing cells but not in GFP or S89A cells, suggesting that the phospho-S89 antibody recognizes phosphorylated ARID3B (Figure 3A). Western blot for total ARID3B confirms the expression of WT and S89A in the cell extracts. Then, we utilized the phospho-S89 antibody to analyze S89 phosphorylation on endogenous ARID3B in subcellular fractions of OVCA429 cells. Western blot analysis detected a ~60 kD band specifically in the chromatin-bound fraction in the endogenous ARID3B protein (Figure 3B). Blotting for Histone H3 (nuclear chromatin bound) and Integrin b1 (membrane) demonstrated successful fractionation. Next, we conducted immunofluorescence (IF) on multiple cell lines using the ARID3B S89 antibody. IF confirmed nuclear localization of phospho-S89 in OVCA429, OVCA433, OVCAR4, and U87MG cells (Figure 4). The figure contains IF for S89, DAPI staining for nuclei, and merged GFP and DAPI. The bottom panels contain IF for total ARID3B (and DAPI counterstain). Total ARID3B IF demonstrated nuclear localization of ARID3B in addition to membrane localization in OVCA433 cells (as previously reported).

**Figure 5:**
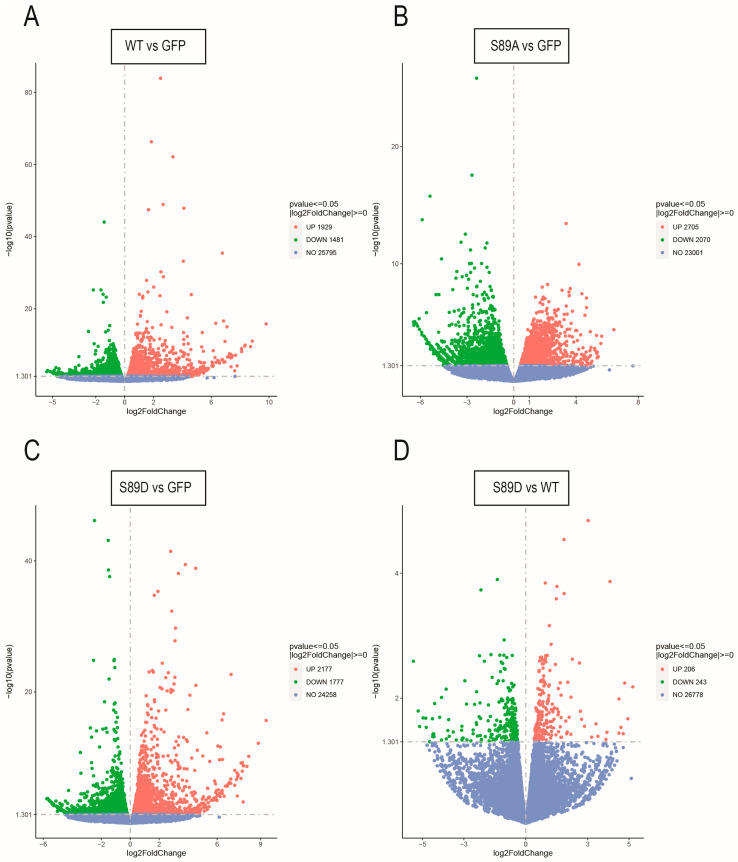
Expression of ARID3B and ARID3B phosphorylation mutants results in altered gene expression profiles. RNA-sequencing was conducted on 3 independent collections of RNA from OVCA429 cells expressing GFP, WT, S89A, and S89D. Volcano plots illustrate differential gene expression in OVCA429 cells expressing ARID3B constructs relative to controls. Comparisons include WT ARID3B-GFP versus GFP (**A**), S89A and GFP (**B**), S89D and GFP (**C**), and S89D and WT (**D**). Upregulated genes are shown in red, downregulated genes in green, and genes with no significant change in blue. Dashed lines divide induced versus repressed genes.

Given that WT ARID3B is phosphorylated and that both WT and S89D localize predominantly to the nucleus, we hypothesized that WT and S89D would induce similar transcriptional programs. In contrast, we predicted that S89A would exhibit altered or diminished transcriptional activity due to reduced nuclear localization. To test these predictions, we performed RNA-sequencing (RNA-seq) analysis on cells expressing GFP, WT ARID3B, ARID3B S89A, and ARID3B S89D. Volcano plots demonstrate the number of genes altered between each of the OVCA429 cells expressing ARID3B constructs and the GFP control cells (Figure 5). As expected, overexpression of WT ARID3B or the two S89 mutants results in over a thousand genes induced or repressed. Comparing gene expression between WT and S89A, there are almost 1900 significantly differentially expressed genes. Similarly, comparing gene expression between S89D and S89A, there are almost 1800 differentially expressed genes. However, when comparing WT versus S89D cells, we observe significant changes in fewer than 500 genes (Figure 5D). Notably, analysis of differential gene expression between ARID3B variants and GFP control cells using the GO Biological Processes database revealed that WT and S89D shared nine of their top 10 significantly enriched pathways (Table 1), whereas no overlap was observed with S89A. Similar results were obtained querying the KEGG database, where WT and S89D shared 7/10 of their most significantly enriched pathways, whereas S89A shared only one (Supplementary Table S2). The RNA-seq data demonstrate that WT and S89D share similar transcriptional activities, which are not shared with S89A.

**Figure 6:**
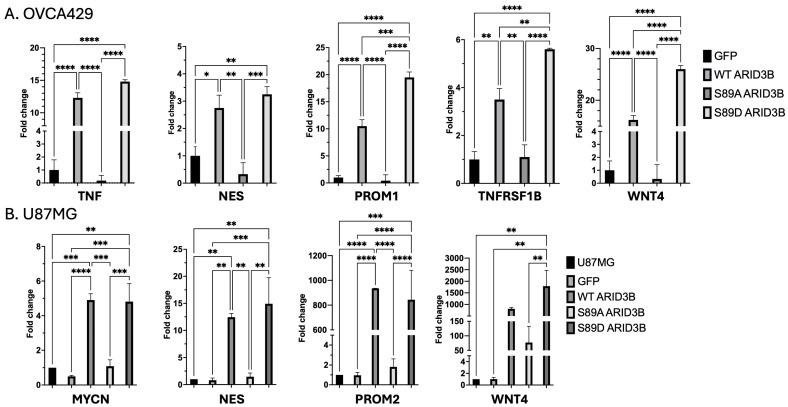
ARID3B S89 phosphorylation modulates expression of ARID3B target genes. RT–qPCR analysis of ARID3B target gene expression in OVCA429 and U87MG cells lines. (**A**) RNA collected from OVCA429 cell lines expressing GFP, or GFP fusions to WT ARID3B, S89A, or S89D. RT-qPCR was conducted for NES, PROM1, TNF, TNFRSF1B, and WNT4. Fold changes are relative to expression in GFP cells. (**B**) RNA collected from Parental U87MG and U87MG cells expressing GFP, or GFP fusions to WT ARID3B, S89A, or S89D. RT-qPCR was conducted for MYCN, NES, PROM2, and WNT4. Fold changes are relative to expression in GFP cells. (**A**,**B**) The delta delta CT method was used for analysis, and gene expression levels were normalized to GAPDH. Data represent the mean ± SEM of 3 biological replicates. *p*-values are indicated: * = *p* < 0.0332, ** = *p* < 0.0021, *** = *p* < 0.0002, and **** = *p* < 0.0001.

In examining the RNA-seq data, we found that about 90% of the genes induced by WT or S89D were previously demonstrated to be ARID3B-regulated. To validate RNA-seq findings, we performed RT-qPCR analysis of selected differentially expressed genes in OVCA429 and U87MG cells (Figure 6). As shown in Supplementary Table S3, we chose to validate known ARID3B target genes that were both highly induced and modestly induced. We also examined ARID3B regulation of PROM1, as it is a direct ARID3B target; however, it did not show a significant change in this RNA-seq data set. In agreement with the RNA-seq data, NES, PROM1, TNF, TNFRSF1B (TNFR2), and WNT4 were significantly induced in WT and S89D-expressing OVCA429 cells. MYCN, NES, PROM2, and WNT4 were all induced in WT and S89D U87MG cells. In OVCA429 cells, NES, PROM1, TNF, and WNT4 were all decreased in cells expressing S89A [12]. The top 50 differentially regulated genes for each comparison are presented in Supplementary Table S4. These data indicate that phosphorylation at S89 is required for full activation of ARID3B-regulated gene expression programs. RT-qPCR was conducted on four independent transductions and RNA isolations.

**Figure 7:**
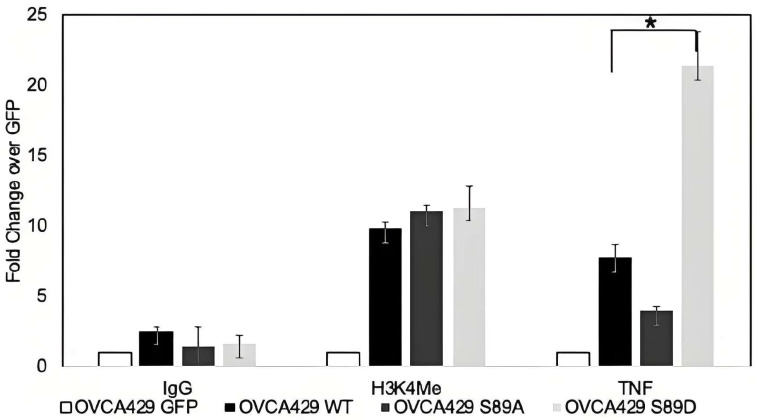
Chromatin immunoprecipitation analysis of ARID3B DNA binding. Chromatin immunoprecipitation (ChIP), followed by quantitative PCR, was performed to assess binding of ARID3B wildtype (WT), S89A, and S89D at the TNF locus. Enrichment is shown as fold change over GFP following normalization to input DNA. IgG and H3K4me served as negative and positive ChIP controls, respectively. WT and S89D demonstrated increased association with the target gene compared to GFP and IgG. WT, S89A, and S89D each showed significantly increased binding relative to GFP (* = *p* < 0.05). The S89A mutant exhibited reduced binding compared to the D mutant (*p* = 0.0236). Data represent the mean ± SEM of 3 biological replicates.

Chromatin immunoprecipitation (ChIP) assays were conducted since phospho-ARID3B localizes to chromatin. We crosslinked chromatin and protein in OVCA429 cells expressing GFP, WT, S89A, or S89D. We determined if ARID3B or the phosphorylation mutants were directly associated with the DNA consensus site from TNF. We chose to use the ARID3B binding site in TNF at chr17:79917005–79917199, since TNF is highly induced by WT and S89D in OVCA429 cells (RNA-seq and Figure 6). ChIP-qPCR demonstrated that WT and S89D exhibited increased binding to the TNF gene, whereas S89A showed reduced chromatin association compared with WT or S89D (Figure 7). ChIP was conducted on three different isolations of chromatin. These findings link S89 phosphorylation to enhanced chromatin binding and transcriptional regulation.

**Figure 8:**
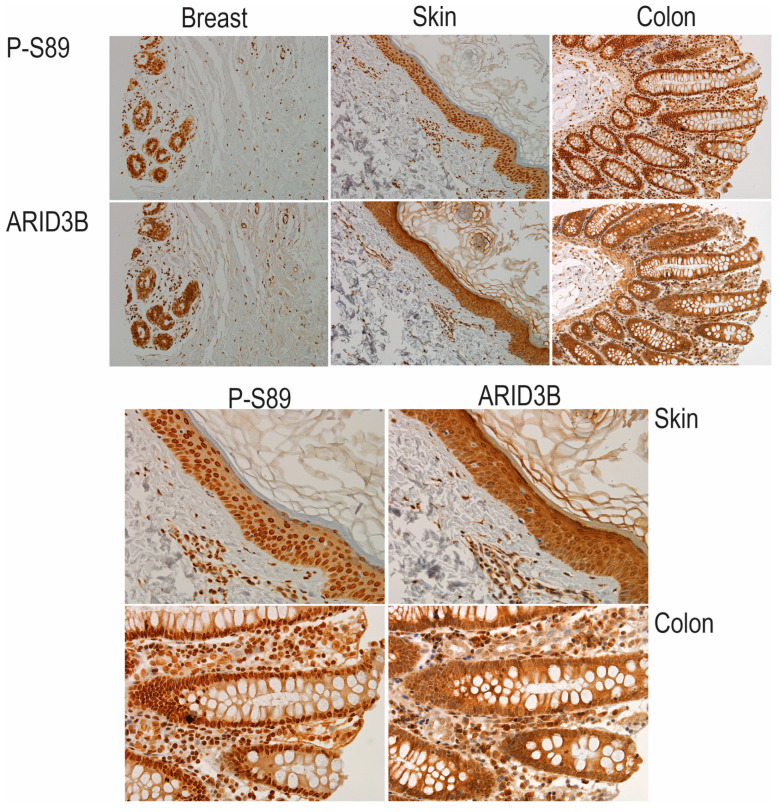
Immunohistochemical detection of phosphorylated and total ARID3B in normal human tissues. Immunohistochemistry (IHC) was performed on serial sections of normal human tissues using antibodies against P-S89 ARID3B and total ARID3B, allowing direct comparison of localization and expression patterns. Brown indicates positive staining. Blue is hematoxylin (nuclei).

Finally, to assess the relevance of ARID3B phosphorylation in human tissues, we performed immunohistochemistry (IHC) for phospho-S89 ARID3B on commercially available tissue microarrays. Previous studies demonstrated that ARID3B is overexpressed in cancer and a variety of human tissues [5,8]. Notably, ARID3B is highly expressed in squamous epithelium (skin, esophagus, and cervix), colon, and breast tissue [8,10]. IHC demonstrated prominent nuclear localization of phospho-S89 in skin, cervix, colon, and breast tissue. Not surprisingly, total ARID3B protein is also detected in these tissues (Figure 8); however, there is greater cytoplasmic ARID3B staining when using the antibody that recognizes amino acids 100–150 of ARID3B (total ARID3B). The data suggest differential localization of phosphorylated versus total ARID3B.

## 4. Discussion

In this study, we identify phosphorylation of ARID3B at serine 89 as a previously unrecognized regulatory mechanism that governs both its subcellular localization and, consequently, transcriptional activity. Although ARID3B has been described as an exclusively nuclear transcription factor, accumulating evidence suggests that ARID3B can also localize to the cytoplasm and membrane. The underlying basis for these differences remained unclear. Our findings provide a mechanistic explanation for this variability, demonstrating that S89 phosphorylation functions as a molecular switch that promotes nuclear retention, chromatin association, and activation of ARID3B-dependent transcriptional programs.

There are numerous ways that post-translational modifications (PTMs), such as phosphorylation, impact the function of transcription factors. Phosphorylation adds a negative charge that may alter the tertiary and quaternary structure of ARID3B and ARID3B-containing complexes. PTMs often regulate transcription factor function by altering DNA-binding activity and interactions with other proteins, including co-factors [13]. PTMs also control transcription factors through the regulation of nuclear import. NFkB and estrogen receptor signaling are prime examples. We observed that the phospho-dead S89A mutant retains the ability to enter the nucleus, yet accumulates substantially in the cytoplasm (Figure 1 and Figure 4). This data implies that S89 phosphorylation may primarily regulate nuclear retention and chromatin engagement rather than initial nuclear entry. Endogenous phospho-S89 ARID3B is enriched in chromatin fractions (Figure 3B) and not in the cytoplasm. This supports a model where phosphorylation of ARID3B is critical for directing its concentration in the nucleus.

Our evidence supports the hypothesis that phosphorylation of ARID3B regulates nuclear localization of the protein and thereby alters its ability to regulate gene expression. The evidence supporting a phosphorylation-dependent model of ARID3B regulation is multifaceted. First, both WT ARID3B and the phosphomimetic, S89D, localize robustly to the nucleus across all cell types examined, whereas the phosphopho-dead S89A mutant displays substantial cytoplasmic distribution (Figure 1, Figure 2, Figure 3 and Figure 4). Importantly, WT and S89D also show similar transcriptional profiles and similar enrichment at chromatin-bound ARID3B target genes, strongly indicating that phosphorylation is required for full transcriptional competence (Figure 5, Figure 6 and Figure 7). Second, our development of a phospho-specific antibody enabled direct visualization of endogenous phospho-S89, revealing that phosphorylated ARID3B is not only nuclear but preferentially enriched within the chromatin fraction (Figure 3B and Figure 4). These findings suggest that phosphorylation enhances nuclear import/retention and chromatin engagement.

Our data add an important new dimension to the current understanding of ARID3B regulation. Prior studies focused primarily on post-transcriptional control of ARID3B abundance by the let-7 and miR-125a miRNAs [6]. While such mechanisms likely contribute to ARID3B overexpression in cancer, they do not explain how ARID3B activity is modulated once the protein is expressed. Our study shows that PTM through phosphorylation is a critical determinant of ARID3B’s functional output, providing a complementary mechanism that may work in concert with miRNA networks in tumors. Moreover, although S89A is capable of entering the nucleus, impaired nuclear recruitment and/or retention results in diminished activation of target genes.

More broadly, our findings place ARID3B within a well-established paradigm in which transcription factor activity is regulated through PTM. Phosphorylation, acetylation, ubiquitination, and SUMOylation are widely used mechanisms to modulate transcription factor stability, localization, DNA-binding affinity, and interactions with co-regulatory proteins. In fact, the ARID3B paralogs, ARID3A and ARID3C, are relegated to lipid rafts via a SUMOylation site that is absent in ARID3B [14]. Different modifications allow rapid and reversible control of transcriptional activity in response to cellular signaling events. The identification of S89 phosphorylation, therefore, reveals an additional regulatory layer that fine-tunes ARID3B function independently of mechanisms controlling its abundance. The post-translational control may be particularly important in cancer cells where ARID3B expression is elevated, providing a means to modulate the transcriptional output of the protein even when expression levels remain high.

Understanding ARID3B phosphorylation helps reconcile discrepancies regarding ARID3B localization in both normal and cancer tissues. Our immunohistochemistry data using the phospho-specific antibody showed that nuclear ARID3B was enriched specifically when phosphorylated at S89, whereas total ARID3B staining reveals additional cytoplasmic protein (Figure 8). These observations implied that a tissue-specific kinase or phosphatase activity may determine ARID3B‘s subcellular distribution. Identifying the upstream kinase(s) responsible for S89 phosphorylation is an important future direction. Defining the kinase/phosphatase regulatory axis will be essential to fully understand how ARID3B activity is modulated across normal and malignant tissues. Using in silico approaches, several kinase families are possible candidates for S89 phosphorylation. These include CDK1/2/5, MAPKs, CK2, PKA/PKC family, and GSK3. Future studies will identify the kinase responsible for ARID3B phosphorylation and determine if the distribution of the kinase in tissues contributes to the differential subcellular localization of ARID3B in cancer.

Our work has implications for cancer biology. ARID3B is overexpressed in multiple tumors and promotes cancer stem cell expansion and tumor growth. The present data show that when ARID3B is phosphorylated, it is more effective at inducing target genes. If validated in vivo, this mechanism may represent a targetable vulnerability in tumors that depend on ARID3B-driven transcriptional programs. Pharmacologic inhibition of the responsible kinase may shift ARID3B into a less active cytoplasmic state, thereby suppressing cancer stem cell traits. Additionally, detection of phospho-S89 in tissues could serve as a biomarker for ARID3B activation status and help stratify patients who may benefit from therapies targeting this pathway.

In summary, we identified phosphorylation of ARID3B at serine 89 as a central regulatory mechanism that determines its nuclear localization, chromatin association, and transcriptional activity. These findings reshape our understanding of ARID3B biology, reveal a new layer of regulation of cancer stem cell programs, and uncover a promising therapeutic target. Continued investigation into the upstream signaling pathways controlling ARID3B phosphorylation will provide valuable insight into how cancer cells regulate stemness and may open new avenues for therapeutic intervention.

## Figures and Tables

**Table 1:** Pathway Enrichment Analysis: GO biological processes.

**GFP vs. WT**	**Term_Name**	**Z Score**	***p* Value**
	cellular response to type I interferon (GO:0071357)	33.29	1.69 × 10^−29^
	type I interferon signaling pathway (GO:0060337)	33.29	1.69 × 10^−29^
	cytokine-mediated signaling pathway (GO:0019221)	5.78	2.61 × 10^−28^
	negative regulation of viral life cycle (GO:1903901)	21.33	7.23 × 10^−19^
	negative regulation of viral genome replication (GO:0045071)	22.72	9.27 × 10^−17^
	endoplasmic reticulum lumen (GO:0005788)	6.17	1.41 × 10^−15^
	interferon-gamma-mediated signaling pathway (GO:0060333)	15.06	5.06 × 10^−15^
	regulation of viral genome replication (GO:0045069)	16.15	9.84 × 10^−15^
	neutrophil degranulation (GO:0043312)	4.34	2.33 × 10^−14^
	neutrophil activation involved in immune response (GO:0002283)	4.30	3.14 × 10^−14^
**GFP vs. D**	**Term_Name**	**Z Score**	***p* Value**
	cellular response to type I interferon (GO:0071357)	31.20	5.47 × 10^−28^
	type I interferon signaling pathway (GO:0060337)	31.20	5.47 × 10^−28^
	negative regulation of viral life cycle (GO:1903901)	26.52	5.18 × 10^−23^
	cytokine-mediated signaling pathway (GO:0019221)	4.98	1.95 × 10^−22^
	negative regulation of viral genome replication (GO:0045071)	29.44	5.16 × 10^−21^
	regulation of viral genome replication (GO:0045069)	21.84	7.34 × 10^−20^
	lysosome (GO:0005764)	4.88	1.07 × 10^−15^
	neutrophil degranulation (GO:0043312)	4.58	1.13 × 10^−15^
	endoplasmic reticulum lumen (GO:0005788)	6.17	1.41 × 10^−15^
	neutrophil activation involved in immune response (GO:0002283)	4.54	1.56 × 10^−15^
**GFP vs. A**	**Term_Name**	**Z Score**	***p* Value**
	response to endoplasmic reticulum stress (GO:0034976)	7.68	1.12 × 10^−9^
	autophagosome (GO:0005776)	9.20	5.27 × 10^−8^
	intrinsic apoptotic signaling pathway in response to endoplasmic reticulum stress (GO:0070059)	16.27	1.40 × 10^−6^
	vacuole (GO:0005773)	7.63	2.92 × 10^−6^
	autophagosome organization (GO:1905037)	7.59	9.46 × 10^−6^
	autophagosome assembly (GO:0000045)	7.28	1.27 × 10^−5^
	pre-autophagosomal structure (GO:0000407)	13.92	1.67 × 10^−5^
	neutral amino acid transmembrane transporter activity (GO:0015175)	13.15	2.17 × 10^−5^
	negative regulation of intrinsic apoptotic signaling pathway (GO:2001243)	6.73	2.22 × 10^−5^
	mitochondrion disassembly (GO:0061726)	8.92	3.77 × 10^−5^

## Data Availability

The original contributions presented in this study are included in the article/Supplementary Materials. Further inquiries can be directed to the corresponding author.

## Supplementary Materials

The following supporting information can be downloaded at: https://www.mdpi.com/article/10.3390/cells15070612/s1. Supplementary Table S1 is a list of primers used in RT-qPCR. Supplementary Table S2 is Pathway Enrichment Analysis of KEGG pathways for RNA-seq. Supplementary Table S3 is a list of gene identified as differentially expressed on RNA-Seq that were validated via RT-qPCR. Supplementary Table S4 contains a list of the top 50 induced genes identified in RNA-Seq compared to GFP control cells for WT, S89D and S89A. Figure S1 is the quantitation of subcellular localization of ARID3B-GFP constructs.

## Abbreviations

The following abbreviations are used in this manuscript:

ARID3B	AT-rich interaction domain–containing protein 3B
ARID3A	AT-rich interaction domain–containing protein 3A
WT	Wild-type
S89	Serine 89
S89A	Serine 89 to alanine mutant (phospho-dead)
S89D	Serine 89 to aspartic acid mutant (phosphomimetic)
Phospho-S89	Phosphorylated ARID3B at serine 89
GFP	Green fluorescent protein
SDM	Site-directed mutagenesis
IP	Immunoprecipitation
IB	Immunoblot
ChIP	Chromatin immunoprecipitation
IF	Immunofluorescence
IHC	Immunohistochemistry
RNA-seq	RNA sequencing
RT-qPCR	Reverse transcription quantitative polymerase chain reaction
EGFR	Epidermal growth factor receptor
NES	Nestin
PROM1	Prominin-1 (CD133)
PROM2	Prominin-2
TNF	Tumor necrosis factor
TNFR2	Tumor necrosis factor receptor 2/TNFRSF1B
WNT4	Wingless-type MMTV integration site family member 4
MYCN	MYCN proto-oncogene
CSCs	Cancer stem cells
PTM	Post-translational modifications
