# Supplementary material for: Serine 89 Phosphorylation Controls Nuclear Localization and Transcriptional Activity of ARID3B
Source: Cells. 2026 Mar 30;15(7):612. doi: 10.3390/cells15070612 (PMC13072255; doi:10.3390/cells15070612)
Supplement: Supplementary file 1 [file cells-15-00612-s001.zip › cells-4181839-supplementary.pdf]

Supplementary Table S1:
Primers for RT-qPCR

NCBI Gene Symbol	IDT Assay ID	Transcript	Location
GAPDH	Hs.PT.39a.22214836	NM_002046(1)	exon 2-3
MYCN	Hs.PT.58.23025106	NM_005378(1)	exon 2-3
NES	Hs.PT.58.40894423	NM_006617	exon 1-2
PROM1	Hs.PT.58.40132695	NM_001145847(7)	exon 20-22
PROM2	Hs.PT.58.39205589	NM_001165977(3)	exon 10-11
TNF	Hs.PT.58.45380900	NM_000594(1)	exon 1b - 4a
TNFRSF1B	Hs.PT.58.40638488	NM_001066	exon 2-3
WNT4	Hs.PT.58.19601929	NM_030761(1)	exon 1-2

Supplementary Table S2. Pathway Enrichment Analysis: KEGG Pathways			
**GFP vs WT**	**term_name**	**zscore**	**pvalue**
	Interferon alpha/beta signaling Homo sapiens R-HSA-909733	30.72	8.63E-29
	Cytokine Signaling in Immune system Homo sapiens R-HSA-1280215	5.71	2.39E-27
	Interferon Signaling Homo sapiens R-HSA-913531	11.15	3.71E-26
	Immune System Homo sapiens R-HSA-168256	3.41	1.35E-22
	Extracellular matrix organization Homo sapiens R-HSA-1474244	5.84	6.04E-15
	Interferon gamma signaling Homo sapiens R-HSA-877300	11.83	1.05E-14
	Herpes simplex infection Homo sapiens hsa05168	6.06	4.33E-11
	Type II interferon signaling (IFNG) Homo sapiens WP619	19.16	5.49E-11
	Influenza A Homo sapiens hsa05164	5.51	3.03E-09
	Innate Immune System Homo sapiens R-HSA-168249	2.75	3.55E-09
**GFP vs D**	**term_name**	**zscore**	**pvalue**
	Interferon Signaling Homo sapiens R-HSA-913531	11.15	3.71E-26
	Interferon alpha/beta signaling Homo sapiens R-HSA-909733	27.09	7.29E-26
	Cytokine Signaling in Immune system Homo sapiens R-HSA-1280215	4.90	1.61E-21
	Immune System Homo sapiens R-HSA-168256	3.24	1.72E-20
	Extracellular matrix organization Homo sapiens R-HSA-1474244	6.25	1.60E-16
	Interferon gamma signaling Homo sapiens R-HSA-877300	11.09	1.23E-13
	Lysosome Homo sapiens hsa04142	8.34	3.50E-12
	Type II interferon signaling (IFNG) Homo sapiens WP619	19.16	5.49E-11
	Antigen processing and presentation Homo sapiens hsa04612	8.89	6.18E-09
	Senescence and Autophagy in Cancer Homo sapiens WP615	7.21	7.42E-09
**GFP vs A**	**term_name**	**zscore**	**pvalue**
	Cytosolic tRNA aminoacylation Homo sapiens R-HSA-379716	13.15	2.17E-05
	Macroautophagy Homo sapiens R-HSA-1632852	6.14	4.19E-05
	Unfolded Protein Response (UPR) Homo sapiens R-HSA-381119	5.22	5.63E-05
	tRNA Aminoacylation Homo sapiens R-HSA-379724	7.90	7.40E-05
	Arginine and proline metabolism Homo sapiens hsa00330	6.42	2.31E-04
	Senescence and Autophagy in Cancer Homo sapiens WP615	4.17	3.01E-04
	IRE1alpha activates chaperones Homo sapiens R-HSA-381070	5.75	4.21E-04
	Transcriptional misregulation in cancer Homo sapiens hsa05202	3.09	6.19E-04
	Aminoacyl-tRNA biosynthesis Homo sapiens hsa00970	4.68	1.28E-03
	Interferon alpha/beta signaling Homo sapiens R-HSA-909733	4.52	1.52E-03

Supplementary Table S3: Genes selected for validation:

	GFP vs WT	GFP vs S89A	GFP vs S89D
Gene	logFC	logFC	logFC
MYCN	0.628	2.65	0.719
NES	0.709	-2.69	0.544
PROM2	0.27	-1.4	0.288
TNF	3.8		3.577
TNFRSF1B	3.8	-1.74	3.77
WNT4	4.51	0.766	4.33

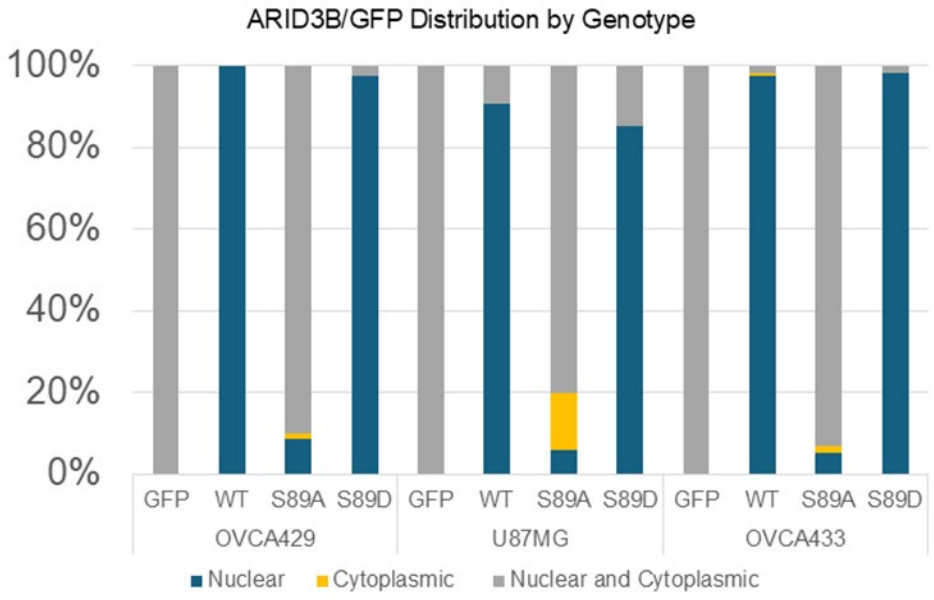

Supplementary Figure S1: Quantitation of subcellular localization of ARID3B-GFP constructs in live cells.

Live cell microscopy was conducted on OVCA429, U87MG, and OVCA433 cells expressing GFP alone, wild-type (WT) ARID3B–GFP, ARID3B–GFP S89A, or ARID3B–GFP S89D. The total number of cells for 3 fields on 3 slides were counted. The number of cells with all nuclear, all cytoplasmic, or both nuclear and cytoplasmic distribution of the constructs were counted.

Supplementary Table S4 : Top 50 induced genes compared to GFP control cells

vs. WT ARID3B			vs ARID3B S89D			vs ARID3B S89A		
gene_symbol	*logFC*	*P.Value*	*gene_symbol*	*logFC*	*P.Value*	*gene_symbol*	*logFC*	*P.Value*
CDKN1C	4.89	4.55E-10	CDKN1C	4.39	2.02E-09	NUPR1	5.72	1.25E-04
CRB2	4.79	2.18E-08	AL138799.1	4.37	2.43E-08	DDIT3	5.44	2.44E-06
AL138799.1	4.54	2.31E-08	CRB2	4.37	6.59E-08	KLHDC7B	4.88	8.33E-06
WNT4	4.51	1.78E-08	WNT4	4.33	2.51E-08	HMOX1	4.12	3.92E-06
CSF1	4.49	2.95E-15	CSF1	4.15	1.10E-14	GDF15	3.97	4.48E-05
PDZK1IP1	4.34	1.52E-13	IFI27	4.15	3.49E-09	LURAP1L	3.89	1.52E-06
RASSF4	4.33	3.54E-13	PDZK1IP1	4.13	2.68E-13	LCN2	3.84	2.25E-04
MRAS	4.24	2.16E-06	RASSF4	4.02	9.21E-13	KRT81	3.70	5.77E-04
IFI27	4.05	5.28E-09	ACVRL1	3.95	1.67E-03	OSGIN1	3.59	3.49E-04
ACVRL1	4.00	1.58E-03	MRAS	3.86	6.22E-06	AL138799.1	3.47	5.70E-04
EDN2	3.88	9.49E-10	TNFRSF1B	3.78	3.57E-10	HERPUD1	3.37	2.47E-06
TNFRSF1B	3.80	3.51E-10	TNF	3.58	4.12E-06	INHBE	3.34	1.18E-05
TNF	3.80	2.34E-06	SIK1	3.48	3.44E-07	TNFSF18	3.32	2.45E-04
GNAO1	3.75	1.56E-06	GNAO1	3.47	3.81E-06	SLPI	3.31	2.00E-03
S100A9	3.59	3.66E-07	S100A9	3.44	6.13E-07	TRIB3	3.26	5.63E-05
LY6D	3.54	2.07E-05	OAS2	3.41	5.43E-14	FTH1P2	3.17	3.19E-04
ARID3B	3.50	5.74E-15	EDN2	3.39	5.32E-09	BEX2	3.16	1.25E-04
INHBB	3.50	5.32E-08	LAMP3	3.36	1.20E-07	CHAC1	3.16	2.66E-05
HSD11B2	3.32	4.00E-10	ARID3B	3.36	8.83E-15	PPP1R15A	3.13	1.65E-05
IVL	3.22	2.31E-05	LY6D	3.35	2.79E-05	LMNTD2	3.11	2.31E-03
BGN	3.19	2.68E-12	HSD11B2	3.13	1.21E-09	AGR2	3.10	3.22E-04
MAB21L4	3.18	1.98E-09	IFI6	3.10	4.05E-11	VLDLR-AS1	3.10	1.16E-02
CEACAM1	3.15	1.59E-07	AQP1	3.06	4.42E-08	CDC42EP5	3.08	2.57E-03
AQP1	3.09	3.95E-08	TNNI2	3.04	8.30E-03	ABTB1	3.08	1.99E-04
OAS2	3.08	2.72E-13	BGN	3.03	5.05E-12	AC104563.1	3.08	5.73E-03
MMP19	3.07	1.03E-06	LBH	3.00	5.92E-07	VPS37D	3.00	1.21E-03
TNNI2	3.05	8.45E-03	MMP19	2.99	2.05E-06	TMEM150A	3.00	1.78E-04
IFI6	3.04	6.52E-11	INHBB	2.97	8.06E-07	ROBO3	2.95	2.68E-04
LAMP3	3.01	4.70E-07	MAB21L4	2.90	5.28E-09	SAA1	2.94	1.45E-03
ALPG	2.99	1.57E-07	MX1	2.90	6.27E-15	CA11	2.94	1.15E-03
MYO7B	2.94	1.91E-06	IFITM1	2.87	1.80E-08	FAM83F	2.94	1.17E-01
FBLN1	2.92	6.10E-09	CEACAM1	2.87	6.47E-07	HSPA5	2.94	9.16E-06
CYTH4	2.86	4.49E-08	CHI3L2	2.85	3.80E-08	C1orf54	2.93	2.76E-03
FOXO6	2.82	1.29E-05	IVL	2.78	7.80E-05	SNHG11	2.91	4.63E-04
TRAF1	2.80	8.64E-10	IL2RB	2.72	6.28E-06	C20orf96	2.90	8.54E-05
CSF2	2.79	2.76E-06	PTGIR	2.68	3.46E-03	AC138150.2	2.89	1.47E-03
EFNB1	2.76	3.99E-10	MYO7B	2.64	6.81E-06	LRRC75B	2.89	7.59E-04
CLU	2.74	2.16E-12	CSF2	2.62	7.32E-06	JOSD2	2.87	5.59E-03
ZDHHC1	2.73	5.30E-07	CLU	2.59	4.78E-12	MAFG-DT	2.86	2.86E-03
IL32	2.70	4.02E-13	FBLN1	2.52	4.61E-08	REEP6	2.85	5.50E-04
C20orf204	2.69	1.11E-05	IL1RN	2.51	6.61E-09	HES2	2.85	4.18E-05
TNXB	2.69	2.08E-07	HSD11B1	2.48	3.19E-07	RAB3IL1	2.84	1.61E-04
MX1	2.68	4.09E-14	GNG7	2.46	1.52E-06	MEIS3	2.84	1.65E-03
IL1RN	2.65	3.36E-09	IL32	2.42	1.44E-12	ARID3B	2.84	8.53E-04
IL2RB	2.65	1.73E-05	KCNK3	2.42	6.74E-07	FAM90A1	2.83	7.12E-05
HMOX1	2.62	2.14E-12	OAS1	2.41	5.40E-13	OSCAR	2.83	2.75E-04
KCNK3	2.58	3.29E-07	C20orf204	2.39	4.62E-05	RNF208	2.82	4.49E-03
PAK6	2.56	1.47E-07	VTCN1	2.39	2.21E-03	LGALS9	2.79	9.07E-04
LBH	2.54	3.33E-06	HMOX1	2.39	1.61E-12	BBC3	2.78	2.47E-04
SIK1	2.54	3.52E-05	IFI44L	2.39	5.13E-12	PDZK1IP1	2.77	1.33E-03
